# A pilot study on EORTC or PERCIST for the prediction of progression-free survival with nivolumab therapy in advanced or metastatic gastric cancers: A STROBE-compliant article

**DOI:** 10.1097/MD.0000000000025494

**Published:** 2021-04-16

**Authors:** Masatoyo Nakajo, Kazuhiro Kitajima, Akira Toriihara, Takaaki Arigami, Hiromitsu Daisaki, Akira Nakamura, Takao Ohtsuka, Hiroto Miwa, Takashi Yoshiura

**Affiliations:** a Department of Radiology, Kagoshima University, Graduate School of Medical and Dental Sciences, 8-35-1 Sakuragaoka, Kagoshima 890-8544; b Division of Nuclear Medicine and PET Center, Department of Radiology, Hyogo College of Medicine, 1–1 Mukogawa-cho, Nishinomiya, Hyogo 663-8501; c Department of Radiology, Asahi General Hospital, I 1326 Asahi City, Chiba 289-2511; d Department of Digestive Surgery, Breast and Thyroid Surgery, Kagoshima University, Graduate School of Medical and Dental Sciences, 8-35-1 Sakuragaoka, Kagoshima 890-8544; e Department of Radiological Technology, Gunma Prefectural College of Health Sciences, 323-1, Kamioki-machi, Maebashi, Gunma 371-0052; f Department of Medical Oncology, Asahi General Hospital, I 1326 Asahi City, Chiba 289-2511; g Division of Gastroenterology and Hepatology, Department of Internal Medicine, Hyogo College of Medicine, 1-1 Mukogawa-cho, Nishinomiya, Hyogo 663-8501 Japan.

**Keywords:** ^18^F-FDG-PET/CT, EORTC, gastric cancer, immune checkpoint inhibitors, PERCIST

## Abstract

Recent breakthrough results from immune checkpoint inhibitors (ICIs) have paved the way to a new era of cancer immunotherapy, and have thus led to a paradigm shift of cancer treatment. In particular, inhibition of the antiprogrammed death-1 (PD-1)/programmed death-ligand 1 (PD-L1) axis with ICI, including nivolumab and pembrolizumab, has been emerging as a novel treatment strategy for advanced gastric cancers. An accurate noninvasive assessment of the response to ICI is important for the management of patients with advanced or metastatic gastric cancer.

To examine whether the European Organization for Research and Treatment of Cancer (EORTC) and PET Response Criteria in Solid Tumors (PERCIST) are valuable for predicting progression-free survival (PFS) in patients with advanced or metastatic gastric cancers treated with nivolumab.

Six patients with advanced or metastatic gastric cancers who underwent ^18^F-FDG-PET/computed tomography (CT) scans before, and from 2 to 6 months after initiation of nivolumab therapy between September 2017 and August 2019, were evaluated retrospectively. The correlation between tumor progression and EORTC or PERCIST was assessed with the Fisher's exact test. The PFS was assessed with the Kaplan–Meier method.

Two patients were alive without progression, and the remaining 4 patients exhibited tumor progression. Two patients without progression were classified as partial metabolic response (PMR) patients based on EORTC or PERCIST, while the other 4 patients with progression were classified as progressive metabolic disease (PMD) patients based on EORTC (*P* = .067), or stable metabolic disease (SMD) patients, or PMD patients based on PERCIST (*P* = .067).

The mean and median PFS of all patients was 12.7 months (95% confidence interval [CI], 4.9–20.4 months) and 5 months (95%CI, 4.0–11.0 months). Two EORTC or PERCIST PMR patients showed significantly longer median PFS compared with 4 non-PMR patients (not reached vs 4.0 months, *P* = .044). Three PERCIST PMR or SMD patients also showed significantly longer median PFS compared with 3 PMD patients (not reached vs 4.0 months, *P* = .022). These results suggest that EORTC or PERCIST has the potential to predict PFS of patients with advanced or metastatic gastric cancers treated by nivolumab and further studies are needed to determine its value in larger study populations.

## 1 Introduction

Gastric cancer is the second leading cause of cancer-related deaths and the sixth most frequent cancer worldwide.^[1]^ Currently, curative resection with or without perioperative chemotherapy is a standard treatment for gastric cancer, while for unresectable or metastatic advanced gastric cancer, chemotherapy using drugs, such as platinum compounds, fluoropyrimidines, docetaxel, paclitaxel, and irinotecan are standard therapies.^[2–5]^

Recent breakthrough results from immune checkpoint inhibitors (ICIs), such as the anticytotoxic T-lymphocyte antigen 4 mAb (ipilimumab) and antiprogrammed death-1 (PD-1) mAbs (nivolumab and pembrolizumab) have paved the way to a new era of cancer immunotherapy, and have thus led to a paradigm shift of cancer treatment.^[6–9]^ In particular, inhibition of the PD-1/programmed death-ligand 1 (PD-L1) axis with ICI, including nivolumab and pembrolizumab, has been emerging as a novel treatment strategy for advanced gastric cancers.^[10,11]^ The ATTRACTION-2 study treated patients with unresectable advanced or recurrent gastric cancer with nivolumab and proved a prolonged overall survival (OS) that led to the establishment of standard options for the treatment for advanced gastric cancer based on the Japanese guidelines. However, although anti-PD-1 mAb is a promising approach for advanced gastric cancer patients, the response rate is still limited.^[12]^ Therefore, an accurate noninvasive assessment of the response to ICI is important for the management of patients with advanced or metastatic gastric cancer.

The assessment of therapeutic response is usually based on morphological changes, and the Response Evaluation Criteria in Solid Tumors (RECIST, version 1.1) are the most commonly used criteria to assess the tumor response.^[13]^ However, the measurements of the longest diameter of lesions on computed tomography (CT) in patients with gastrointestinal tumors are not always possible.

The uptake of the glucose analog 2-deoxy-2-[^18^F]fluoro-D-glucose (^18^F-FDG) represents the metabolic activity of glucose, and is extensively used as a tracer of positron emission tomography (PET)/CT in oncology.^[14]^^18^F-FDG-PET/CT-based criteria have also been developed to evaluate the response to therapy, and 2 criteria presently exist for ^18^F-FDG-PET/CT response evaluations:

a) the European Organization for Research and Treatment of Cancer (EORTC) and
b) the PET Response Criteria in Solid Tumors (PERCIST).^[15,16]^

Several groups are exploring the use of ^18^F-FDG-PET scans for the prediction of the responses to ICI therapy.^[17]^ However, to the best of our knowledge, no prior studies have evaluated the usefulness of ^18^F-FDG-PET/CT monitoring with EORTC or PERCIST for patients with advanced or metastatic gastric cancer treated by nivolumab.

The present study was performed to examine whether EORTC or PERCIST is valuable for the prediction of progression-free survival (PFS) of patients with advanced or metastatic gastric cancers who were treated by nivolumab.

## 2 Materials and methods

### 2.1 Patients

From January 2017 to August 2019, 111 patients who underwent nivolumab therapy as systemic treatment for advanced or metastatic gastric cancers in 3 institutions were retrospectively evaluated in this study. The appropriate review board at each institution (Ethics committee on epidemiological studies, Kagoshima University Graduate School of Medical and Dental Sciences, The ethics review board of Hyogo College of Medicine and Institutional review board of Asahi General Hospital) approved the study and waived the requirement for patient informed consent. Clinical records were reviewed to identify patients for analysis.

The following inclusion criteria were used:

1) pathologically proven, locally advanced or metastatic gastric cancers, irrespective of the histologic subtype;
2) patients who underwent both baseline and follow-up ^18^F-FDG-PET/CT scans with the same scanner at the same institution with the schedule described next.

^18^F-FDG-PET/CT scans were performed within 2 months before, and from 2 to 9 months after the initiation of nivolumab therapy. The exclusion criteria were the history or coexistence of other malignancies and treatment with other ICIs before or during nivolumab therapy.

All patients were staged according to the clinical tumor, nodes, and metastases (TNM) classification of the American Joint Committee on Cancer.^[18]^ When available, the pretreatment of the PD-L1 tumor expression was recorded.

### 2.2 ^18^F-FDG-PET/CT examinations

The following 3 whole-body PET/CT scanners were used: Discovery MI (GE Healthcare, WI) at 2 institutions, and Gemini GXL16 (Philips Medical Systems, Eindhoven, The Netherlands) and Discovery iQ HD (GE Healthcare, WI) at the other 1 institution. Patients were instructed to fast for at least 5 hours before the examinations. This resulted in a mean plasma glucose level of 99 mg/dL (range, 83–126 mg/dL) immediately before injection of ^18^F-FDG (216 MBq ± 36 [range, 169–279 MBq]). Static emission images were obtained approximately 60 minutes after injection. Attenuation-corrected PET images were reconstructed with an ordered-subset expectation maximization, iterative reconstruction algorithm, or a Bayesian penalized, likelihood reconstruction algorithm. The acquisition and reconstruction parameters were harmonized to minimize standardized uptake value (SUV) differences between scanners and kept them within the reference range proposed by the Japanese Society of Nuclear Medicine.

Two experienced radiologists who knew the study purpose but were blinded to clinical and pathological information of each patient interpreted the PET/CT images, and they confirmed (after consensus) that the lesions had abnormally increased uptakes with more intense compared with the background uptake.^[19]^

The following quantitative analyses were performed by the third radiologist according to the interpreted results based on visual assessment. The radiologist placed the volume of interest (VOI) manually on a suitable reference fused axial image, defined manually the craniocaudal and mediolateral extent that encompassed the entire target lesion, and then excluded any avid normal structures to obtain the maximum SUVmax. To perform PERCIST, the following 2 short extra steps were needed. The first step was the measurement of the normal liver, mean SUV normalized lean body mass (SULmean) that was completed quickly based on a VOI (with a diameter of 3 cm), that was placed in the right lobe of the liver. The next step was the calculation of the peak SUV (SUVpeak). It was automatically calculated in a spherical VOI (diameter of 1.2 cm) that was automatically placed on the hottest site of the tumor above the manually drown tumor VOI, and then was normalized to the lean body mass (SULpeak). The free software package RAVAT (Nihon Medi-Physics Co., Ltd. Tokyo, Japan) calculated the SUVmax and SULpeak automatically. The reduction percentage of SUVmax and SULpeak ([baseline value − follow-up value] × 100%/baseline value) were then calculated manually.

### 2.3 EORTC and PERCIST

The responses to nivolumab were classified as complete metabolic response (CMR), partial metabolic response (PMR), stable metabolic disease (SMD), or progressive metabolic disease (PMD).

Tumor responses according to EORTC were as follows.^[15]^ The CMR was defined as a complete resolution of ^18^F-FDG uptake within the measurable target lesion so that it was indistinguishable from the surrounding background with no new ^18^F-FDG-avid lesions. In patients with metabolically active lesions on the follow-up scans, the SUVmax values of the lesions (up to a total of 5) of the baseline and follow-up scans were summed (maximum of 2 per organ). If the sum of the SUVmax values decreased by at least 25%, the tumor response was classified as PMR. PMD denoted a 25% increase of the sum of the SUVmax values or the detection of new ^18^F-FDG-avid lesions that are characteristic of cancer. The SMD is a disease other than CMR, PMR, or PMD.

PERCIST requires that

1) the difference between baseline and follow-up normal liver SULmean values must be within 20% (and <0.3 SUL mean units) to be assessed, and
2) the SULpeak of the tumor had to be greater than the threshold which was defined equal to 1.5 times that of mean liver SUL value + 2 standard deviations (SDs) (mean liver activity).^[16]^

This threshold value was also quickly and automatically calculated with the software. If the tumor SULpeak at baseline did not exceed this threefold value, the patient was not eligible for response evaluation with PERCIST.

Tumor responses according to PERCIST were assessed by 2 different approaches.^[19]^ The first approach was PERCIST5, and this analysis was performed in a manner that was almost the same as that described for EORTC5, but the sum of the SULpeak values was used. Given that the hottest lesions were selected in each scan, target lesions on follow-up scans were not necessarily the same as the target lesions at baseline. CMR completely characterized the ^18^F-FDG uptake within the measurable target lesion to less than the mean liver activity, and was indistinguishable from the surrounding background with no new ^18^F-FDG-avid lesions. PMR was defined as the decrease of the sum of the SULpeak value by at least 30%. PMD was defined as an increase of the sum of the SULpeak by at least 30% or in the cases of new ^18^F-FDG-avid lesions. SMD includes diseases other than CMR, PMR, or PMD. The second approach was the immunotherapy-modified PERCIST5 (imPERCIST5). Although the definition of PMD was different from PERCIST5, the other criterion was the same as PERCIST5. In imPERCIST5, the appearance of new lesions alone did not result in PMD, and PMD was defined only by an increase of the sums of the SULpeak values by 30%. New lesions were included in the sum of the SULpeak if they showed higher uptake than existing target lesions, or if fewer than 5 target lesions were detected on the baseline scan.

### 2.4 Follow-up of patients

Medical records provided information on patient prognoses. The last follow-up was conducted in May 2020. Disease progression was established by clinical and imaging follow-up information. If treatment was maintained at the 6-month follow-up, patients were considered to have a durable clinical benefit (DCB) of immunotherapy, as done in previous studies.^[20,21]^ PFS was the period from the start of nivolumab to the date of disease progression, death, or the last follow-up, whichever occurred first. Patients who were alive without recurrence or metastasis at the time of the last follow-up were treated as censored.

### 2.5 Statistical analyses

The Fisher's exact test was used to compare categorical data. Survival curves were drawn using the Kaplan–Meier method and the significant difference between survival curves was tested with the log-rank test.

Data are presented as mean values with SDs. A value of *P* < .05 was considered as statistically significant and all *P* values presented were two-sided. The MedCalc Statistical Software (MedCalc Software, Mariakerke, Belgium) was used for statistical analyses.

## 3 Results

### 3.1 Patient characteristics

Of the total of the 111 patients studied herein, 76 patients were excluded because neither baseline nor follow-up ^18^F-FDG-PET/CT scans were performed. Nineteen patients were excluded because they only had pretreatment ^18^F-FDG-PET/CT scans. Another 6 patients were also excluded because only underwent post-treatment ^18^F-FDG-PET/CT scans. Four patients were excluded because other ICIs were administrated before nivolumab therapy.

Finally, 6 patients (2 males, 4 females; mean [±SD] age, 71 ± 11 years; range, 59–90 years) were eligible for the analyses. The characteristics of the patients are summarized in Table 1. Three patients showed advanced gastric cancer, and 2 of them showed 2 lung or 2 lymph node metastases, respectively. The remaining 3 patients showed postoperative recurrence with peritoneal or bone metastasis, respectively. Regarding the TNM stage, 1 patient was in stage IIIC and 5 in stage IV.

**Table 1:** Characteristics of 6 patients with advanced or metastatic gastric cancers.

No.	Sex	Age (yr)	Status	Metastatic lesions	Stage	Number of nivolumab course at follow-up PET/CT	Follow-up PET/CT duration from initial nivolumab (d)	Reduction percentage of SUVmax	Reduction percentage of SULpeak	New lesion	EORTC	PERCIST		DCB	Clinical course
												PERCIST5	imPERCIST5		
1	F	74	Advanced	Lung	IV	4	58	72.9%	72.2%	No appearance	PMR	PMR	PMR	+	No progression
2	F	90	Advanced	Lymph node	IV	4	60	48.9%	48.3%	No appearance	PMR	PMR	PMR	−	No progression
3	M	68	Recurrence	Peritoneum	IV	11	149	−27.7%	−21.7%	No appearance	PMD	SMD	SMD	+	Progression
4	F	74	Recurrence	Bone	IV	7	123	−130.5%	−112.8%	Adrenal gland	PMD	PMD	PMD	+	Progression
5	F	61	Recurrence	Peritoneum	IV	9	125	−178.1%	−255.0%	Peritoneum	PMD	PMD	PMD	−	Died
6	M	59	Advanced	No appearance	IIIC	10	152	−93.6%	−83.1%	Bone	PMD	PMD	PMD	−	Died

The mean (±SD) interval between the baseline ^18^F-FDG-PET/CT scan and the initiation of nivolumab was 29 ± 27 days (range 1–60 days). All patients received nivolumab (240 mg) every 2 weeks as immunotherapy. The nivolumab therapy was performed in the second (n = 2) or third lines (n = 4) of treatment. The pretreatments of the PD-L1 tumor expression were missing in all patients.

### 3.2 EORTC or PERCIST (PERCIST5 and imPERCIST5) with survival prediction

Three primary lesions and 7 metastatic lesions were visible on baseline ^18^F-FDG-PET/CT. The follow-up ^18^F-FDG-PET/CT scans completed after 4 to 11 courses of nivolumab therapy [4 courses (n = 2), 7 courses (n = 1), 9 courses (n = 1), 10 courses (n = 1), and 11 courses (n = 1)], and the mean (±SD) interval between initiation of nivolumab and follow-up ^18^F-FDG-PET/CT scans was 111 ± 42 days (range 58–152 days).

On follow-up ^18^F-FDG-PET/CT, 2 patients achieved PMR by EORTC or both approaches of PERCIST (PERCIST5 and imPERCIST5). The reduction percentages of SUVmax in these 2 patients were 72.9% and 48.9%, and the reduction percentages of the SULpeak values were 72.2% and 48.3%, respectively. One patient presented a SMD that was detected by both approaches of PERCIST, but PMD was detected only by EORTC. The reduction percentage of SUVmax and SULpeak in this patient was −27.7% and −21.7%, respectively. In the remaining 3 patients, new lesions became apparent on the follow-up ^18^F-FDG-PET/CT scan; 1 patient with an adrenal metastasis, 1 patient with a peritoneal metastasis, and 1 patient with a bone metastasis. Thus, responses were classified as PMD by either EORTC or PERCIST5, and these 3 patients were also classified as PMD by imPERCIST5.

The median duration of the follow-up was 18.0 (range 5–29) months. Among the patients with PMR, 1 patient reached a DCB, but treatment was stopped after 4 courses of nivolumab in the other patient owing to immune-related colitis. These 2 patients were alive without progression during the follow-up period.

Conversely, even if 1 patient with SMD detected by PERCIST (PMD detected by EORTC) reached a DCB, the patient eventually showed tumor progression after 7 months of follow-up ^18^F-FDG-PET/CT scans. One patient with PMD also reached a DCB with continuous treatment for 29 months, but the metastatic lesion showed progression. The treatment was stopped in the remaining 2 patients with PMD owing to tumor progression, and did not reach DCB. These 2 patients died after 5 and 9 months of initiation of nivolumab.

There was no significant relationship between DCB and EORTC or PERCIST (each, EORTC, *P* = 1.00; PERCIST, *P* = 1.00) or between DCB and tumor progression (*P* = 1.00). Two patients without progression were classified as PMR by either EORTC or PERCIST, while 4 patients with progression were classified as PMD by EORTC, or either SMD or PMD by PERCIST. Although each relationship was close to a significance level (alpha = 0.05), there was no significant relationship between tumor progression and EORTC or PERCIST (each, EORTC, *P* = .067; PERCIST, *P* = .067).

The mean and median PFS of all patients was 12.7 months (95% confidence interval [CI], 4.9–20.4 months) and 5 months (95%CI, 4.0–11.0 months), respectively. Although 2 patients with PMR by EORTC or PERCIST showed significantly longer median PFS compared with the other 4 patients with PMD or SMD (not reached [95%CI,notapplicable] vs 4.0 months [95%CI,4.0–11.0months], *P* = .044), these differences were close to a significance level (alpha = 0.05). These 2 patients also showed longer median PFS compared with 1 patient with SMD or the other 3 patients with PMD (not reached [95%CI, not applicable] vs 11.0 months [95%CI, not applicable] vs 4.0 months [95%CI, 4.0–5.0 months], *P* = .049) based on PERCIST; however, these differences were also close to a significance level (alpha = 0.05). On the other hand, when the patients were divided into non-PMD (PMR or SMD) and PMD based on PERCIST, 3 non-PMD patients showed significantly longer median PFS compared with the 3 other PMD patients (not reached [95%CI, not applicable] vs 4.0 months [95%CI, 4.0–5.0 months], *P* = .022).

**Figure 1:**
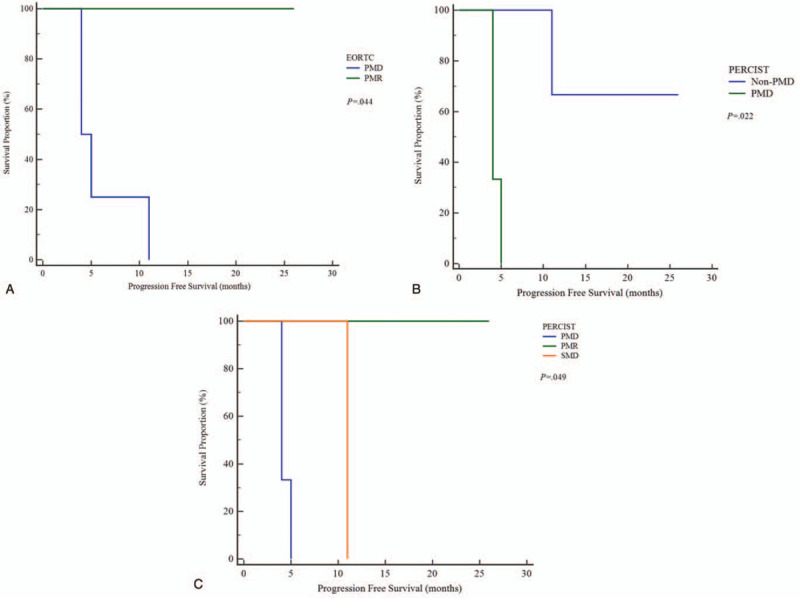
Kaplan–Meier survival curves of the progression-free survival (PFS) in patients with gastric cancer who received nivolumab. Significant differences were demonstrated among EORTC (A) and PERCIST (B: between non-PMD and PMD, C: among PMR, SMD, and PMD) criteria.

**Figure 2:**
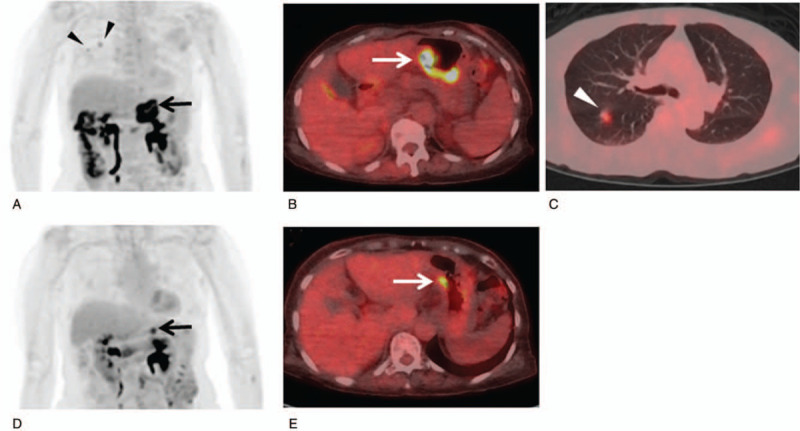
A 74-year-old woman with advanced gastric cancer with lung metastases (stage IV) received nivolumab. Baseline ^18^F-FDG-PET/CT [maximum intensity projection (MIP) (A) and fused transaxial (B and C) images] shows abnormal ^18^F-FDG uptake in the primary lesion (A and B: arrows) and right lung metastases (A and C: arrowheads). The follow-up ^18^F-FDG-PET/CT after 4 courses of nivolumab therapy [MIP (D) and fused transaxial (E) images] show decreases of ^18^F-FDG uptake in the primary lesion (D and E: arrows) with disappearance of lung metastases. The reductions of the sum of SUVmax and SULpeak were 72.9% (15.76–4.27) and 72.2% (9.46–2.63), respectively. The status was PMR according to EORTC or PERCIST. The patient was alive without progression 26 mo after the initiation of nivolumab.

**Figure 3:**
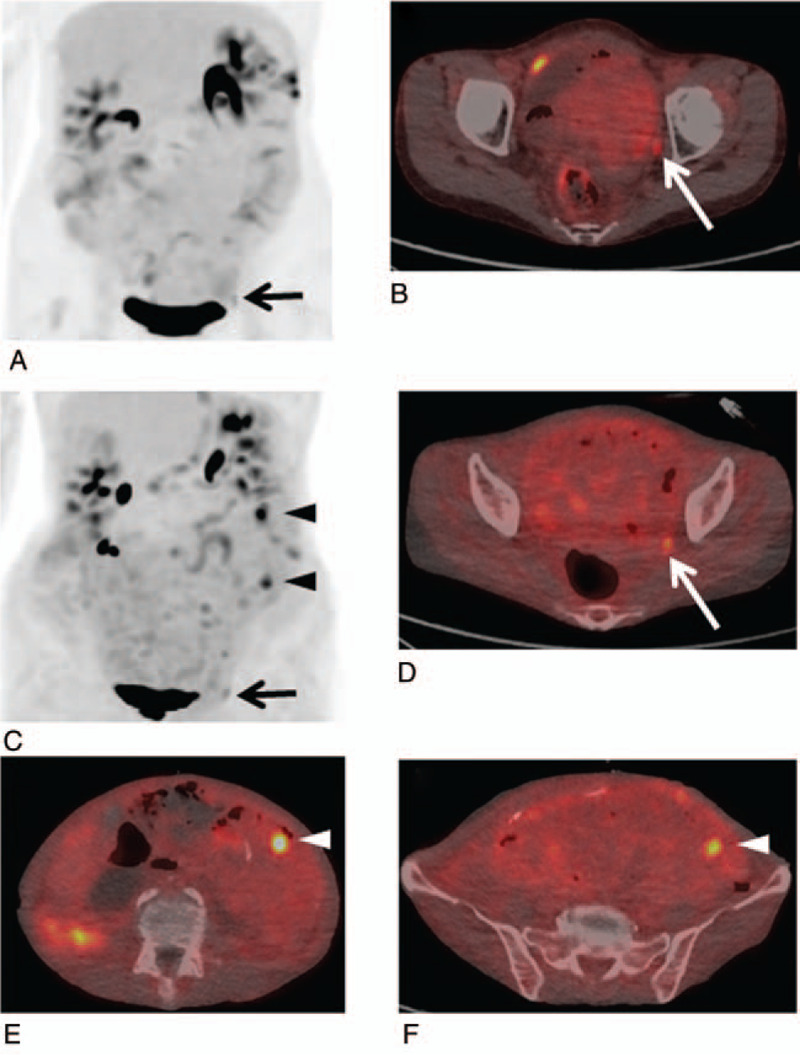
A 61-year-old woman with postoperative recurrence gastric cancer with peritoneal metastasis (stage IV) received nivolumab. Baseline ^18^F-FDG-PET/CT [MIP (A) and fused transaxial (B) images] shows abnormal ^18^F-FDG uptake in the peritoneal metastasis in the left peritoneal cavity (A and B: arrows). The follow-up ^18^F-FDG-PET/CT after 9 courses of nivolumab therapy [MIP (C) and fused transaxial (D–F) images] show the progression of known peritoneal metastasis (C and D: arrows) with new hypermetabolic peritoneal metastases (C, D, and F: arrowheads). The increase of the sum of the SUVmax and SULpeaks were 178.1% (2.65–7.37) and 255.0% (1.42–5.04), respectively. The status was PMD according to EORTC or PERCIST. The patient exhibited progressive disease at 4 mo and died 5 mo after the initiation of nivolumab.

Figure 1 shows the risk differences in PFS between PMR and PMD based on EORTC, and between non-PMD and PMD and among PMR, SMD, and PMD based on PERCIST. The representative ^18^F-FDG-PET/CT images of non-progression and progression cases are shown in Figures 2 and 3, respectively.

## 4 Discussion

In this study, we examined the clinical value of EORTC or PERCIST to predict PFS in patients with advanced or metastatic gastric cancers on nivolumab therapy.

To-this-date, there have been several reports that have examined the usefulness of ^18^F-FDG-PET/CT to monitor tumor response to ICIs. Sachpekidis et al^[22]^ reported that the assessment of tumor response after 2 cycles of ipilimumab according to EORTC criteria was predictive of the final outcome in patients with melanoma. Ito et al^[19]^ evaluated imPERCIST5 for ipilimumab in patients with metastatic melanoma. The patients with responder (CMR or PMR) had a significantly longer 2-year OS compared with those with nonresponders (SMD or PMD). Additionally, imPERCIST5 was an independent prognostic factor for OS at multivariate analysis. Thus, they recommended the use of ^18^F-FDG-PET/CT to assess tumor response ipilimumab in research and clinical trials.

To our knowledge, no studies have previously investigated the ability of EORTC or PERCIST to predict tumor response and prognosis of patients with advanced or metastatic gastric cancer who received ICI. Although EORTC and PERCIST adopt different approaches to achieve tumor response evaluations as mentioned in Section 2, in our study, EORTC and PERCIST agreed on 83.3% (5/6) of the patients. One patient with SMD by PERCIST was categorized as PMD by EORTC. Nasir et al^[23]^ reported that the agreement between EORTC and PERCIST criteria for treatment response evaluation in patients with solid malignant tumors. The same authors mentioned that adoption of EORTC or PERCIST in ^18^F-FDG PET/CT reports can standardize the evaluation of oncological treatment results. In our study, although there was no significant correlation between tumor progression and EORTC or PERCIST (each, EORTC, *P* = .067; PERCIST, *P* = .067) even if each relationship was close to a significance level (alpha = 0.05), the patients with PMR by either EORTC or PERCIST showed significantly longer mean PFS compared with patients without PMR. However, the analyzed patients were only 6 cases, to confirm whether EORTC or PERCIST is useful for the prediction of PFS of patients with advanced or metastatic gastric cancers treated by nivolumab is needed in a much larger population.

This study had the following limitations. First, the results were obtained by a retrospective review of a selected patient group with a small study population. Although the study was conducted at 3 institutions, the sample size included only 6 cases. During the study period, 105 patients were not included for the analysis because neither the baseline nor the follow-up ^18^F-FDG-PET/CT scans were performed, or other ICIs were administrated before nivolumab therapy, even though 111 patients received nivolumab as the systemic treatment for advanced or metastatic gastric cancer in these 3 institutions. Therefore, a prospective study is needed in a much larger population. Second, the Kaplan–Meier method with log-rank test was only applied to examine the relationship between EORTC or PERCIST and PFS, and not examined the relationship among other clinical prognostics factors and PFS. Third, the times of follow-up of the ^18^F-FDG-PET/CT scans were not standardized that may have affected changes in tumor ^18^F-FDG uptake and the number of lesions detected. Forth, 4 different scanners were used. Although the harmonization of SUVs was performed, the variability in SUV measurements may have affected the results of SUVmax and SULpeak. However, in the cases of identical scanning, image reconstruction and data processing conditions were met among the studies of patients, and SUVs seemed to be quite independent of the applied methodology, as was shown by Boellaard et al.^[24]^. Thus, the SUVmax or SULpeak values of the baseline and follow-up scans obtained by the same scanner and protocol can be used to calculate the percent changes in the SUVmax or SULpeak values to assess EORTC or PERCIST.

In conclusion, although the study population was only 6 patients with advanced or metastatic gastric cancers treated by nivolumab, median PFS was significantly longer in the EORTC or PERCIST PMR patients than non-PMR patients and in the PERCIST PMR or SMD patients than PMD patients. These results suggest that EORTC or PERCIST has the potential to predict PFS of patients with advanced or metastatic gastric cancers treated by nivolumab and further studies are needed to determine its value in larger study populations.

## Acknowledgments

We thank Prof. Chihaya Koriyama, who belongs to Department of Epidemiology and Preventive Medicine, Kagoshima University, for the validation of the statistical analysis.

## Author contributions

**Conceptualization:** Masatoyo Nakajo, Kazuhiro Kitajma.

**Data curation:** Masatoyo Nakajo, Kazuhiro Kitajma, Akira Toriihara, Takaaki Arigami, Akira Nakamura, Takao Ohtsuka, Hiroto Miwa.

**Formal analysis:** Masatoyo Nakajo.

**Methodology:** Masatoyo Nakajo, Kazuhiro Kitajma.

**Software:** Hiromitsu Daisaki.

**Supervision:** Kazuhiro Kitajma, Takashi Yoshiura.

**Visualization:** Masatoyo Nakajo, Kazuhiro Kitajma, Akira Toriihara.

**Writing – original draft:** Masatoyo Nakajo.

**Writing – review and editing:** Masatoyo Nakajo, Takashi Yoshiura.
